# Efficient deep learning-based approach for malaria detection using red blood cell smears

**DOI:** 10.1038/s41598-024-63831-0

**Published:** 2024-06-10

**Authors:** Muhammad Mujahid, Furqan Rustam, Rahman Shafique, Elizabeth Caro Montero, Eduardo Silva Alvarado, Isabel de la Torre Diez, Imran Ashraf

**Affiliations:** 1 Artificial Intelligence and Data Analytics (AIDA) Lab, CCIS, Prince Sultan University, 11586 Riyadh, Saudi Arabia; 2 School of Computer Science, University College Dublin, Dublin, D04 V1W8 Ireland; 3 Department of Information and Communication Engineering, Yeungnam University, Gyeongsan, 38541 Republic of Korea; 4 Universidad Europea del Atlantico, 39011 Santander, Spain; 5 Universidad Internacional Iberoamericana Arecibo, Puerto Rico, 00613 USA; 6 Universidade Internacional do Cuanza, Cuito, EN250 Angola; 7 Universidad Internacional Iberoamericana, 24560 Campeche, Mexico; 8 Universidad de La Romana, La Romana, República Dominicana; 9 Department of Signal Theory, Communications and Telematics Engineering, University of Valladolid, 47011 Valladolid, Spain

**Keywords:** Malaria detection, EfficientNet, Transfer learning, Disease detection, Computational biology and bioinformatics, Health care

## Abstract

Malaria is an extremely malignant disease and is caused by the bites of infected female mosquitoes. This disease is not only infectious among humans, but among animals as well. Malaria causes mild symptoms like fever, headache, sweating and vomiting, and muscle discomfort; severe symptoms include coma, seizures, and kidney failure. The timely identification of malaria parasites is a challenging and chaotic endeavor for health staff. An expert technician examines the schematic blood smears of infected red blood cells through a microscope. The conventional methods for identifying malaria are not efficient. Machine learning approaches are effective for simple classification challenges but not for complex tasks. Furthermore, machine learning involves rigorous feature engineering to train the model and detect patterns in the features. On the other hand, deep learning works well with complex tasks and automatically extracts low and high-level features from the images to detect disease. In this paper, EfficientNet, a deep learning-based approach for detecting Malaria, is proposed that uses red blood cell images. Experiments are carried out and performance comparison is made with pre-trained deep learning models. In addition, k-fold cross-validation is also used to substantiate the results of the proposed approach. Experiments show that the proposed approach is 97.57% accurate in detecting Malaria from red blood cell images and can be beneficial practically for medical healthcare staff.

## Introduction

Malaria is a serious public health issue, particularly in the world’s tropical and subtropical climates. According to the 2015 World Health Organization (WHO) report^1^, the Plasmodium parasite caused 405,000 fatalities. Screening for malaria entails using blood slides and a microscope to detect infected red blood cells, which may be a time-consuming and laborious job^2^. Pathologists must analyze a large number of cases, and studies reveal that the bulk of malaria cases occur in Africa (85%), South East Asia (71%), and the Eastern Mediterranean (71%). This high number of cases may have an adverse effect on the quality of malaria screening. Because blood smears are thick and complicated, with numerous cells merged, they can be difficult to interpret. As a blood smear gets contaminated with malaria, the cellular texture changes over time, making it difficult to distinguish between healthy and infected samples. Studying blood smear pictures from numerous perspectives can help diagnose infections more quickly, efficiently, and cost-effectively. Nevertheless, because of increased demand for inspections and a scarcity of pathologists, as well as varying weather and lighting circumstances, this has become a substantial social and economic health issue. To overcome this issue and minimize pathologists’ workload, blood smear slides may now be taken successfully utilizing digital cameras or high-resolution cell phones^3^.

Greater picture quality and directness usually result in more accurate and reliable analysis. Thorough image data, analysis can reveal many complicated features of biological functioning. Portable cell phones are ubiquitous and transformational, offering a low-cost and simple way to quickly capture picture datasets. The quality of blood smear slide photographs obtained with cell phones, on the other hand, is often worse than that of digital cameras. Because of the large number of cases and low-resolution photos, manual interpretation may be difficult, and illness detection using standard machine learning algorithms may be problematic^4^. These challenges can be solved by using quick preprocessing deep learning algorithms that automatically estimate important characteristics for malaria diagnosis and grading^5–8^.

Machine learning methods have lately piqued the interest of academics due to their potential for developing automated malaria diagnosis systems^9,10^. Prior research has used supervised learning algorithms such as support vector machines (SVM), Naive Bayes (NB) classifiers, and neural networks (NN) to detect infections with accuracies ranging from 83.5 to 85%^9,11^. Nevertheless, because these algorithms are very sensitive to the feature extraction approach, it is critical to construct a discriminant feature vector with low redundancy^9,10,12,13^. While effective feature extraction can enhance detection accuracy, the procedure still necessitates human feature vector extraction by qualified professionals, making fully automated diagnosis impossible. To solve this, deep learning methods for malaria cell detection have been developed, with the objective of creating a totally automated diagnosis platform that does not require manual feature extraction.

The identification of malaria parasites is impeded by several limitations, including specific features of blood cell samples, such as their diminutive size and substantial disparity. These challenges present a substantial barrier to attaining precise results, and traditional AI techniques are not suitable for addressing this particular scenario. The objective of this study is to improve the precision and effectiveness of malaria parasite diagnostic methods by creating innovative deep learning-based models for malaria identification. Implementing this strategy would greatly increase the models’ performance, surpassing current benchmarks and resulting in a substantial improvement in malaria diagnosis accuracy. Addressing these critical aspects is essential for enhancing the prognosis of patients with this condition.

Deep learning algorithms may extract hierarchical data representations, with higher layers reflecting increasingly abstract notions that are less sensitive to transformations and scaling^14^. While deep convolutional neural networks have been used to diagnose malaria in thick blood smears, pathologists still struggle to differentiate infected and non-infected samples in thick films because the difference is not as clear as individual red blood cells cropped from whole slide images based on thin films. For simple classification-related tasks, machine learning models perform the best, but for complex tasks, these models cannot provide good accuracy. On the other hand, deep learning addresses complex tasks easily. In this regard, this study presents a deep learning approach for malaria detection and makes the following contributions

- An efficient deep learning model is proposed that detects malaria from red blood cell images accurately and efficiently, while also avoiding overfitting and solving complex problems.
- A comparison of the proposed model is carried out with other fine-tuned deep learning models to validate the efficacy of the proposed model. To evaluate the generalization efficacy of deep learning models, this study employed tenfold cross-validation. Confusion matrix results, training and testing accuracy, and loss of proposed vs. fine-tuned deep learning models are also employed.
- We present a novel, efficient deep learning-based model that comprises a smaller number of layers and is most efficient in terms of performance metrics like accuracy, precision, recall, and f1 score. We also tested the other important deep learning models by adding some supportive layers, fine-tuning the parameters, and then proposing our new model that works superiorly among all other models either used in this study or those already cited in the literature.
- Implementing the proposed model would significantly enhance the accuracy of malaria diagnosis, as it would significantly boost the performance of the models beyond current benchmarks.

The remaining paper is divided into the following sections: “Literature review” section represents the literature review related to malaria detection. “Materials and methods” section describes the materials and methods used in this study. “Results and discussion” section contains the experimental results and discussion. “Conclusion” section presents the conclusion.

## Literature review

Several approaches have been presented regarding malaria detection. Rajaraman et al.^15^ investigated image processing and deep learning techniques to keep up with the most recent advancements in data detection and computer vision for autonomous malaria detection. Over the past decade, an abundance of data has been generated in this extremely active research field. With the advent of digitization, deep learning methods have already had a big impact, and research has produced an exciting and significant innovation. This would render outdated many previously utilized classification techniques. In addition, the overwhelming bulk of these humanly constructed features can be made worthless by deep learning’s introduction of the challenging problem of producing classification features.

The study^16^ presents a CNN-based deep learning model for malaria detection using blood samples. A device is designed where blood samples are smeared and illuminated. The generated images are projected using a mirror and lens for proper focusing. The approximation is later used with the CNN model to determine if the sample is infected or not. Experiments are performed using infected and uninfected samples for malaria. A 97.1% accuracy is reported using the model trained using 1000 epochs. The study^17^ investigates the viability of deep learning models for determining the type of parasitic organisms. Experiments involve VGG19, ResNet50V2, EfficientNetB3, etc. on a large dataset with six classes. A higher classification accuracy of 99% is reported for deep learning models by applying fine-tuning for various parameters.

Soner et al.^18^ employed a deep CNN model for malaria detection from the cell image dataset. They used recurrent neural network (RNN), CNN, and artificial neural network (ANN) models with 27558 images for malaria detection. Because the images vary in width and length, the authors resized them to a fixed $64 \times 64\times 3$ size. The CNN model contained three convolutional layers followed by a max pooling layer, one flattening layer, one hidden layer, and one output layer. They used a 64-bit batch size and 20 epochs to validate the results with a binary loss function. The CNN model trains in 10 min and achieves 97% accuracy on training data and 95% accuracy on testing data. They validated the model’s accuracy with fivefold cross-validation. Similarly^19^, worked on malaria detection and used the same 27,558 cell images dataset for experiments. The authors applied color constancy to all images. The proposed CNN architecture consists of six convolutional layers followed by 8, 16, 32, 64, 128, and 256 filters. They compared the proposed fast CNN model with other deep learning models. They used a variety of features with support vector machines (SVM) to classify the images into infected and uninfected classes. The SVM with a bag of features achieved 85% accuracy, and the proposed model achieved 96% accuracy. The authors utilized images with sizes of $50 \times 50\times 3$ to reduce consumption time and enhance the accuracy of the model.

Along the same lines^20^, classified cell images into infected and uninfected classes using two unique neural networks. The proposed approach worked in three stages: the first was the segmentation of red blood cells (RBC), the second was the cropping and masking of data, and the third was the classification of data into binary classes. On red blood cell images, they attained a 93.75% accuracy. Another study^21^ employed 27,558 single-cell images and three CNN models, including custom CNN, frozen VGG-19 CNN, and fine-tuned VGG-19 CNN, with tenfold cross-validation. The testing accuracy achieved by basic CNN was 94%, frozen CNN was 92%, and fine-tuned CNN was 96%. To accurately detect malaria from red cell images, Zhao et al.^22^ used an automated mobile application with CNN architecture, an object detection model, and up-scaling low-resource images. They classified the balanced dataset into infected and uninfected images with a 96.5% accuracy. The authors in the study^23^ used two CNN-based models, ResNet50 and VGG16 for malaria detection from red blood cell images. The VGG16 attained a 96.15% accuracy, 94.82 sensitivity, and 96.16% F1 score with 2652 true positives, and 2648 true negatives while the ResNet50 model performed poorly. The study^24^ used a deep CNN model to predict malaria from cell images with 95.23% accuracy. The authors used three convolutional and three pooling layers, as well as fully connected ‘ReLU’ and ‘sigmoid’ layers.

A deep learning model is developed in^25^ for malaria detection from microscopic blood images and is reported to obtain a 91% accuracy. Negi et al.^26^, used preprocessing and augmentation approaches to detect malaria in 2021 using the Kaggle cell-images dataset. The images were scaled to $224\times 224\times 3$, and padding and horizontal flipping were used to increase the diversity of the data. After 15 epochs, they had a 95.7% accuracy and a 0.31 loss. Emrah^24^ used a novel CNN model with 20 weighted layers for malaria detection. A total of 27,558 images of thin blood cells were used to train and evaluate the CNN model, which resulted in an overall accuracy of 95.28 percent. The experimental findings on a large medical dataset demonstrate the efficacy of the proposed deep learning methodology for detecting malaria disease.

Maqsood et al.^27^ evaluated the efficacy of various deep learning models for effective malaria detection. In addition, the authors also proposed a modified CNN model that outperforms existing deep learning models. Bilateral filtering and image enhancement techniques are used to identify red blood cell features before training the model. The modified CNN model was generalized and prevented over-fitting owing to image augmentation techniques. The benchmark NIH malaria dataset was used for all experiments and the results show that the proposed method is 96.82% accurate at identifying malaria from small blood smears. A new deep learning model called a “data augmentation convolutional neural network” (DACNN), trained using reinforcement learning, was proposed in^28^. The performance of the proposed DACNN model was compared against that of CNN and directed acyclic graph (DAGCNN) models. The findings show that DACNN outperforms past studies in the processing and classification of images. Its classification accuracy in photos of malaria blood samples from the balanced class dataset was 94.79%. Finding malaria parasites in blood smear images can be done with the help of the proposed model.

Hemachandran et al.^29^ implemented three deep learning algorithms: CNN, MobileNetV2, and ResNet50 to detect malaria cases. Upon comparing the constructed models, conclusions regarding their superiority surfaced. The surrounding environment is a significant factor contributing to malaria’s existence and transmission. In contrast to alternative models, ResNet50 demonstrated superior performance and generated more precise outcomes in malaria disease identification. The National Institutes of Health website provided the compilation, which included a total of 27,558 images. In this collection, there were 13,780 images of parasitized cells and 13,778 images of uninfected cells. Ultimately, in an effort to enhance disease detection, the MobileNetV2 model achieved an astounding 97.06% accuracy rate, surpassing the competition.

## Materials and methods

**Figure 1:**
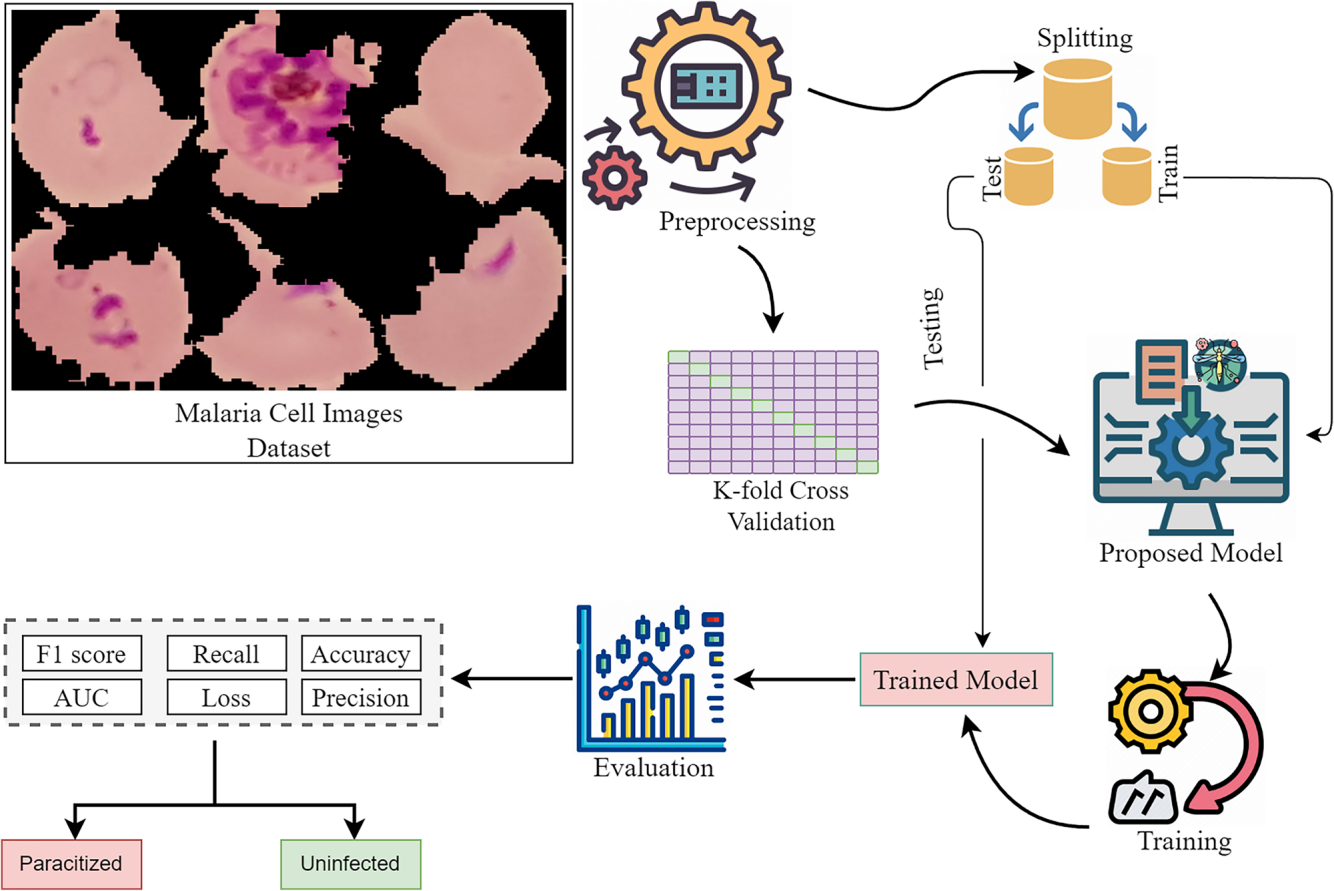
Work flow of proposed methodology.

This section contains the details of the proposed methodology and details of the pre-trained models employed in this study. Figure 1 shows the workflow of the proposed methodology. The details of each step are provided in the subsequent sections.

### Dataset

**Figure 2:**
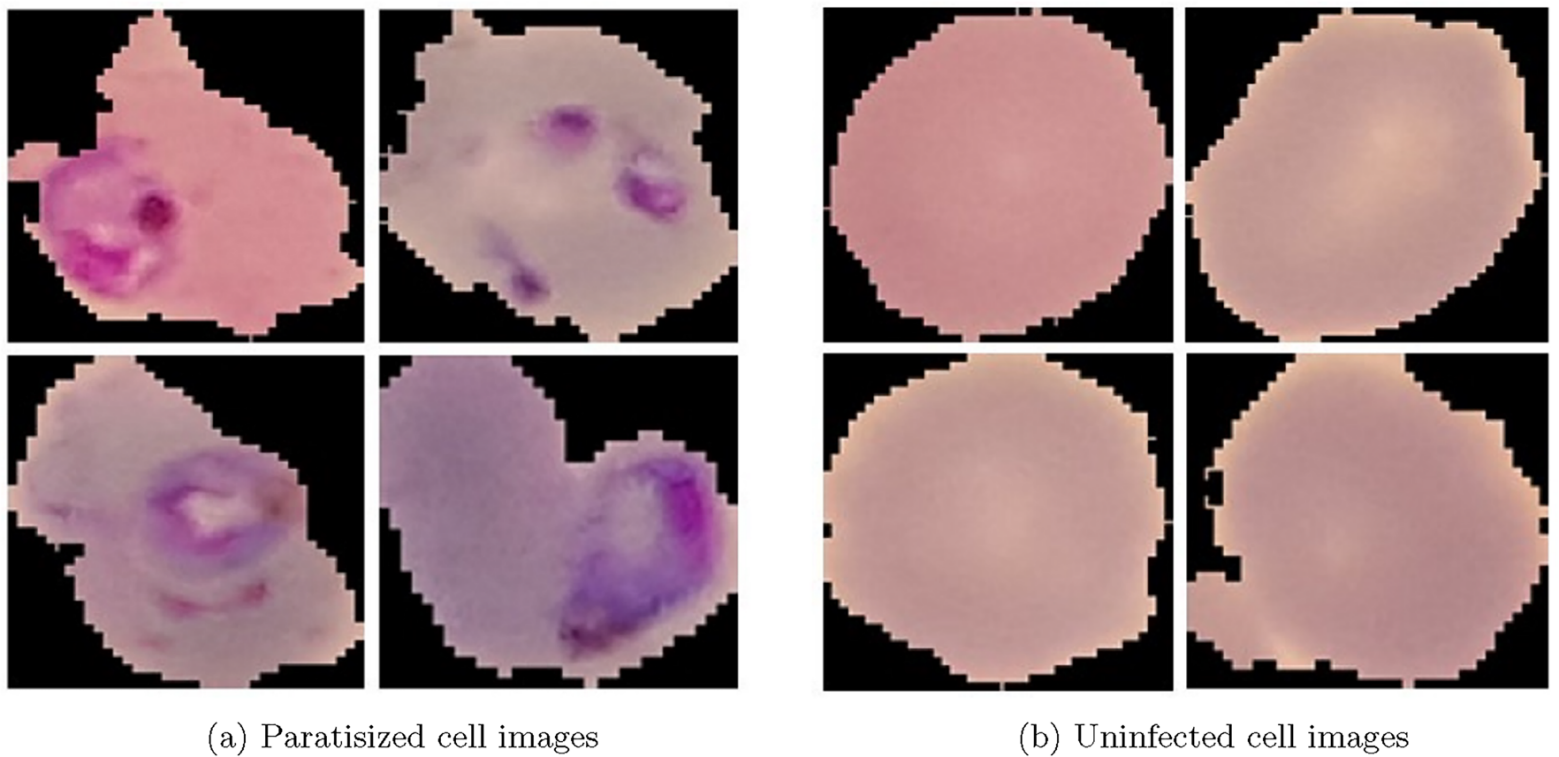
Samples taken from the red blood cell image datasets contain parasitized cell images and uninfected cell images.

The dataset used in this study was obtained from the public data repository. It contains a total of 27,558 cell images with 13,779 parasitized images and 13,779 uninfected images. These images were obtained from 150 unhealthy patients (infected individuals) and 50 healthy patients. The expert slide-readers and pathologists manually annotated the whole dataset. Color variations in red cell images are due to different blood stains during the image acquisition process. Figure 2 shows samples of parasitized cell images and uninfected cell images.

### Preprocessing and data splitting

Preprocessing is a very crucial and initial step for deep learning image classification tasks. The dataset contains 13,779 images of parasitized cells and 13,779 images of uninfected cells, which are equally balanced. The cell images contain various widths and heights, and the deep learning model requires equal or fixed-size input. To test the model’s robustness and compatibility, we resized the images. After resizing, the next important step is to split the cell images into two parts; training and testing. The 80% data are used to train the deep learning models and 20% are kept for testing the model efficacy and performance. Table 1 shows the parasitized and uninfected images after data splitting into train and test.

**Table 1:** Paracitized and uninfected images after data splitting.

Dataset	Paracitized cells	Uninfected cells	Total-images
Training-images	11,023	11,023	22,046
Testing-images	2756	2756	5512
Total_images	13,779	13,779	27,558

### Proposed deep learning model

Deep learning models learn complex patterns of data through various layers. Deep learning has demonstrated effectiveness in many image classification tasks in medical, engineering, and other applications. Deep learning models work well on large datasets, however, consume a lot of computational resources. The hyperparameter settings, loss function, and other layers are used to solve these problems by fastening the training process of deep learning models, reducing computational time, reducing layers, and creating efficient deep models^30^. Transfer learning is a popular technique that favors the pre-trained models that have been trained on large datasets such as ImageNet, and produces better results for small datasets (Table 2).

**Figure 3:**
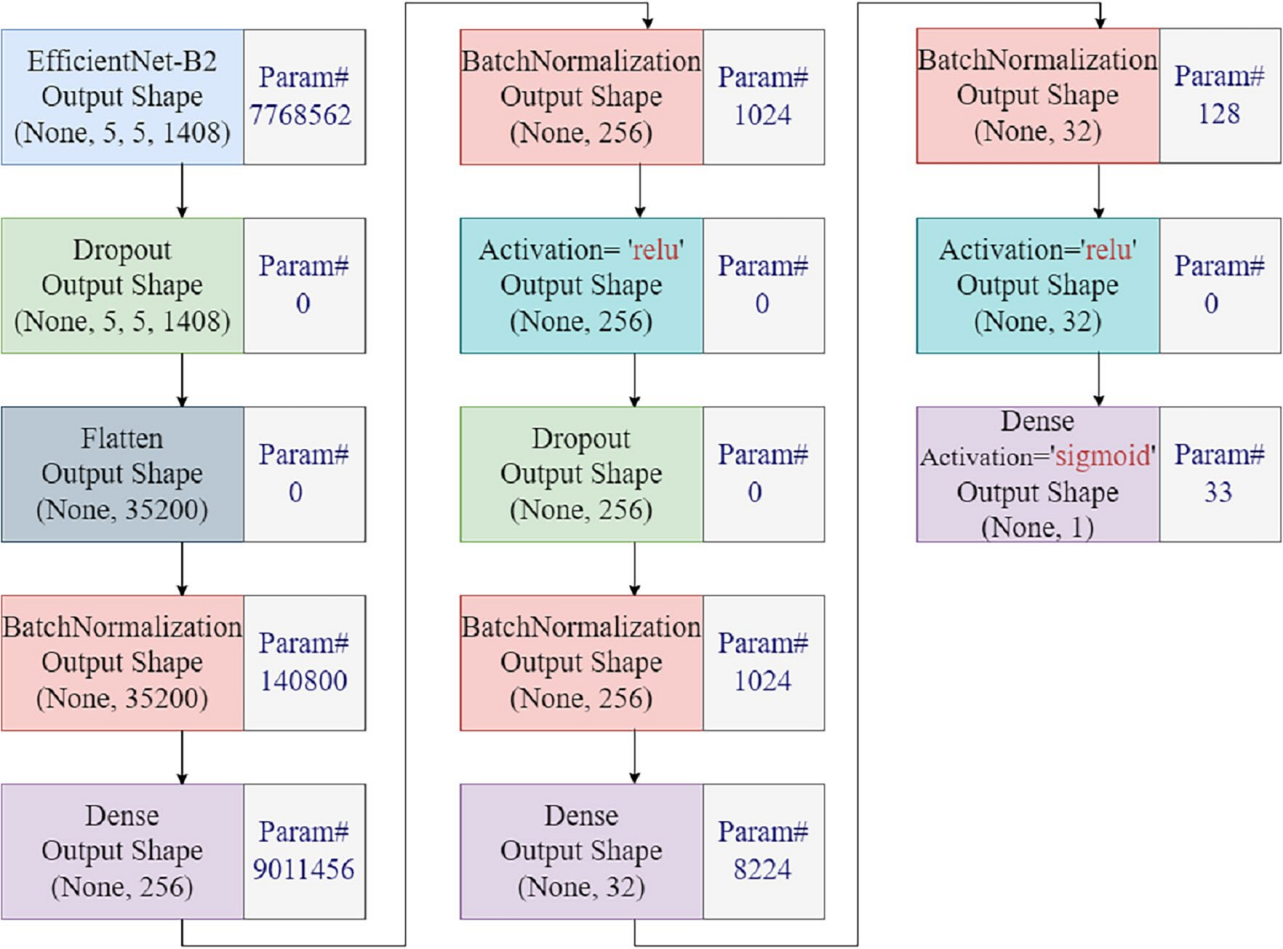
Architecture of proposed deep learning model for malaria detection.

**Table 2:** The number of layers, types, output-shape and its parameters for the proposed model.

Number of layers	Layer type	Output shape	Parameters
1	Efficientnet-B2	None, 5, 5, 1408	7,768,562
2	Dropout	None, 5, 5, 1408	0
3	Flatten	35,200	0
4	Batch normalization	35,200	140,800
5	Dense	256	9,011,456
6	Batch normalization	256	1024
7	Activation	256	0
8	Dropout	256	0
9	Batch normalization	256	1024
10	Dense	32	8224
11	Batch normalization	32	128
12	Activation	32	0
13	Dense	1	33

Total parameters: 16,931,251.

Trainable parameters: 16,792,195.

Non-trainable parameters: 139,056.

EfficientNet-B2 is a CNN model that is exceptionally accurate and reliable and is mostly used for image classification problems. It is well suited for problems that require fewer parameters and have minimal processing resources. Using depth-wise separable convolutions (DWSC), an efficient scaling approach, this model improves the classification accuracy. The main aim of using EfficientNet-B2 in disease detection is its efficiency and accuracy because of its small model size and minimal computing resources. Figure 3 shows the architecture of the proposed model. The development of the EfficientNet-B2 model leads to the addition of a dropout layer, ultimately yielding an output shape of (5,5,1408). We use the flattened layer to convert the multi-dimensional input layer into a one-dimensional one. After that, we utilized three dense layers, four batch normalization layers, and three activation layers. We achieved this after flattening the layers into a single dimension. The first two dense layers of the network utilize ReLU activation functions. The Rectified Linear Unit (ReLU) functions not only collect complicated patterns correctly, but they also lower the chances of overfitting and generalization errors. This makes the model work better overall. The last dense layer primarily employs the sigmoid activation function for classification activities, particularly in binary classification situations. We use this function to complete classification tasks. Batch normalization is an essential component of deep learning architectures that improves accuracy while simultaneously speeding up the training process.

For training purposes, batch normalization uses a small amount of data to calculate the mean and standard deviation of each feature. The statistical data is then used to standardize the input when that step is completed. This approach minimizes internal co-variate shift, which is the change in the distribution of network activation resulting from differences in the parameters of the training process so that it can be used more efficiently. The efficiency of optimization techniques can be increased by batch normalization, which involves standardizing the input. If this is done, the model can be built more quickly and is less likely to encounter gradients that are evaporating or exploding. Additionally, it acts as a regularizer, which means it reduces the need for additional methods of regularization.

Malaria can be detected by analyzing images for symptoms using deep learning models that focus on red blood cells. The proposed model is trained to identify malaria-related symptoms by employing a collection of expert classifications applied to blood cells. Once the model has been adequately trained, it will have the ability to evaluate recently obtained blood cells and offer medical personnel useful information, thereby enabling a faster and more precise diagnosis. Once the model is adequately trained, it possesses the potential to aid physicians in the diagnostic process by classifying newly obtained blood cell samples as either infected or uninfected with malaria. Utilizing deep learning-based malaria detection models in clinical settings offers several potential advantages. These devices have the capability to deliver precise and prompt diagnosis, particularly in regions where there is a scarcity of skilled microscopists. These techniques expedite the initiation of medication for individuals with malaria, enabling front-line healthcare professionals to promptly identify the infection. Consequently, the incidence and mortality rates linked to malaria decline. Moreover, automated analysis is capable of efficiently managing a significant volume of samples on a broad scale, therefore alleviating the workload of laboratory personnel, particularly during outbreaks or monitoring initiatives.

### Pretrained models

This study also employed fine-tuned deep learning models such as CNN, VGG-16, DenseNet version 121,169, 201, Inception version 3, etc. for Malaria detection. Different pre-trained fine-tuned deep learning models and their trainable parameters are given in Table 3.

**Table 3:** Models and their trainable parameters used for malaria detection.

Model	Trainable parameters
DenseNet121	74,049
DenseNet201	1,968,129
DenseNet169	6,870,017
CNN	21,460,993
InceptionV3	38,537,217
VGG16	8,390,657
ResNet50	532,801
EfficientNet-B1	1,501,409
EfficientNet-B7	339,137
MobileNet	5,817,473
MobileNetV2	183,169
Proposed model	16,792,195

#### Convolutional neural network

**Figure 4:**
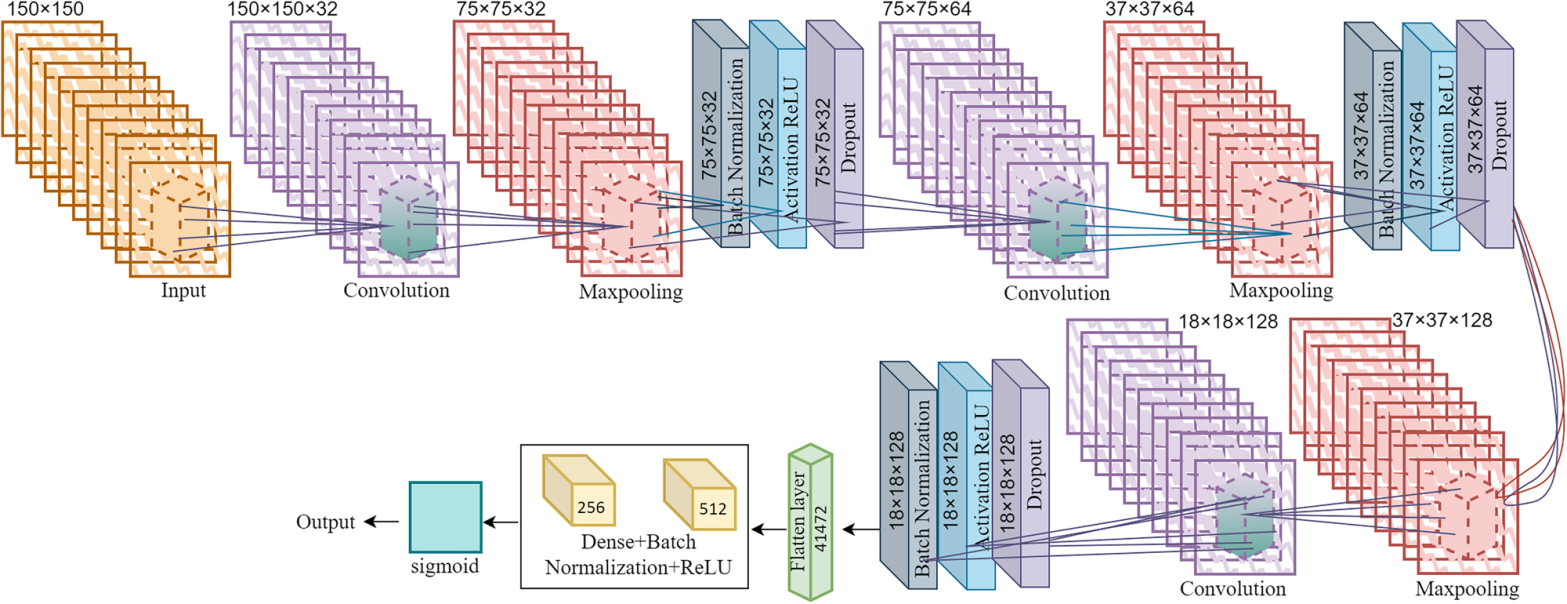
Detailed CNN architecture.

A CNN, a type of neural network, consists of numerous layers and aims to directly identify patterns from image pixels. It requires minimal pre-processing^31^. The convolution layer, the pooling layer, and the fully connected layer are the three essential layers that are widely considered to be the foundation of a CNN. We utilized three convolution blocks, three Maxpooling blocks, and three blocks for Batch normalization, ReLU activation, and Dropout layers. The convolution layer, a fundamental component of a CNN, performs the majority of the computational work. This layer performs the convolution or filtration operation on the input and then transmits the response to the subsequent layer. We place the pooling layer between the successive convolution layers to spatially reduce the input representation and the required processing space. This layer performs the pooling process on each sliced input, thereby reducing the computational workload for the subsequent convolution layer. After that, We flatten all the layers into single dimensions and then add two dense layers with Batch and ReLU activation. The application of the completely linked layer (sigmoid layer) generates the final output, which is also equal to the number of classes^32^. The detailed architecture of CNN is shown in Fig. 4.

#### VGG16

In 2014, VGG16 won the ILSVR (ImageNet) competition and is now considered one of the most advanced vision models available. The VGG-16 network was trained using the ImageNet database and consists of 16 weighted layers, including 13 convolutional layers and 3 fully connected layers. Despite limited image datasets, the VGG-16 network delivers high accuracy due to its extensive training. VGG16 is capable of both object detection and classification with 92.7% accuracy, classifying 1000 images into 1000 unique categories. It is a widely used image classification algorithm that is easy to implement using transfer learning. By adding new layers to neural networks and utilizing batch normalization, the training process can be accelerated, making learning easier and the model more robust^33^.

#### Inception V3

Inception V3 is a deep CNN architecture introduced in 2015 by Google researchers. It is the third version of the Inception family of models and is designed to be more efficient and accurate than its predecessors. The Inception V3 model boasts a more expansive network compared to its predecessors, the Inception V1 and V2 models. This deep CNN is specifically designed to be trained on low-configuration computers, although it is still a challenging process that can take several days. Transfer learning provides a solution to this issue by retaining the parameters of previous layers while only updating the last layer for new categories. This approach involves deconstructing the Inception V3 model by removing its final layer, thereby leveraging the benefits of transfer learning^34^.

#### DenseNet121

DenseNet121 is a CNN architecture that has gained widespread use in image classification tasks since its introduction in 2017. DenseNet121 architecture aims to increase the depth of deep learning networks while improving their training efficiency. This is achieved through the use of short connections between layers. In DenseNet, each layer is connected to all other layers that are deeper in the network, making it a CNN. The number 121 pertains to the count of layers with trainable weights, excluding batch normalization layers. The remaining 5 layers consist of the initial 7 × 7 convolutional layer, 3 transitional layers, and a fully connected layer^35^.

#### DenseNet169

DenseNet169 is a deep CNN architecture that is part of the DenseNet family of models. It was introduced by researchers at Facebook AI Research in 2017 as an improvement over the original DenseNet model. DenseNet169 has 169 layers, which is more than the original DenseNet but less than DenseNet201. Like other DenseNet models, DenseNet169 uses dense connectivity to promote feature reuse and reduce the number of parameters needed to train the network. It also includes bottleneck layers to reduce the computational cost of convolutions. DenseNet169 has achieved state-of-the-art performance on several benchmark datasets, making it a popular choice for image classification tasks requiring high accuracy^36^.

#### DenseNet201

DenseNet201^37^ is a deep CNN architecture. DenseNet201 uses a “dense connectivity” structure, where each layer is connected to every other layer in a feed-forward fashion. This dense connectivity promotes feature reuse and reduces the number of parameters needed to train the network. DenseNet201 also includes a feature called “bottleneck layers” which reduces the computational cost of convolutions by using 1 × 1 convolutions to reduce the dimensionality of the input. DenseNet201 has achieved state-of-the-art performance on several benchmark datasets and is widely used in image classification tasks.

#### ResNet50

ResNet50, an architecture in deep learning, was introduced in 2015 by Microsoft researchers. It has found applications in a range of computer vision tasks, including the analysis of medical images. ResNet50 is designed to overcome the challenge of vanishing gradients by introducing shortcut connections that allow the network to learn residual representations. By utilizing ResNet50, researchers have been able to attain various results in computer vision tasks, including object detection, image classification, and medical image analysis^38^.

#### EfficientNet-B1

EfficientNet-B1 is a neural network architecture that was proposed by Google researchers in 2019. It is part of the EfficientNet family of models that are designed to achieve high accuracy while minimizing computational resources. It has fewer parameters and floating-point operations (FLOP) than larger models but still achieves competitive performance on various benchmark datasets. EfficientNet-B1 has been used in a range of computer vision tasks, including image classification, object detection, and segmentation^39^. Its efficient design makes it particularly suitable for mobile and embedded devices.

#### EfficientNet-B7

EfficientNet-B7 is a powerful model that has shown promising results in a variety of computer vision tasks, including medical image analysis. It is the largest model in the EfficientNet family and has significantly more parameters and FLOP than smaller models in the family. EfficientNet-B7^40^ achieves state-of-the-art performance on various benchmark datasets, including ImageNet, with significantly fewer computational resources than previous state-of-the-art models. However, due to its large size, EfficientNet-B7 may not be suitable for mobile and embedded devices with limited computational resources.

#### MobileNet

MobileNet is a family of neural network architectures that are designed to be efficient on mobile and embedded devices with limited computational resources. It was proposed by Google researchers in 2017 and has since become a popular choice for a range of computer vision tasks. MobileNet achieves its efficiency by using depth-wise separable convolutions, which separate the spatial and channel-wise dimensions of convolutions and reduce the number of parameters and computations. This design allows MobileNet to achieve high accuracy while requiring significantly fewer resources than larger models. MobileNet has been implemented in various frameworks and is widely used in real-world applications^41^.

#### MobileNetV2

MobileNetV2 is a follow-up to the original MobileNet architecture, proposed by Google researchers in 2018. It further improves the efficiency and accuracy of the original architecture by introducing several novel features. One of the key improvements is the use of a bottleneck block that expands and then contracts the number of channels, allowing for better feature extraction. MobileNetV2 also uses a technique called linear bottlenecks, which adds a linear activation function after each depth-wise convolution to further reduce the computational cost. These innovations make MobileNetV2 one of the most efficient neural network architectures for mobile and embedded devices, while still achieving high accuracy on a range of computer vision tasks^39^.

#### Performance measures

The performance of all models that were used in this study was evaluated using precision, recall, F1 score, and accuracy. After training the model, the testing part is used to test the model’s efficiency and classification. The performance is also evaluated using the confusion matrix. The confusion matrix constitutes TP, TN, FP, and FN predictions.

- *TP:* The true positive rate refers to the actual positive class that is predicted to be positive.
- *TN:* The true negative rate refers to the correct negative predictions made by the model among all negative records.
- *FP:* There is a false positive rate that states the actual negative predictions that are classified as positive by the model.
- *FN:* There is a false negative rate that states the records belong to the positive class and are predicted as negative by the model.
- *Accuracy:* The number of truly classified predictions by a model among the total number of predictions it makes or computes to divide the TP plus TN prediction by the total number of predictions.
  1 $$\begin{aligned} = \frac{TP+TN}{TP+TN+FP+FN} \end{aligned}$$
- *Precision:* Precision is the number of true positive predictions from the total number of actual predictions classified by the model or computed to divide the TP predictions by the TP plus FP predictions.
  2 $$\begin{aligned} = \frac{TP}{TP+FP} \end{aligned}$$
- *Recall:* The recall is the score of the correct positive prediction that the model found by looking at all of the actual positive tweets or by dividing the TP predictions by the TP plus FN predictions.
  3 $$\begin{aligned} = \frac{TP}{TP+FN} \end{aligned}$$
- * F1 score:* An F1 score is an evaluation metric that estimates model performance by taking the average of recall and precision.
  4 $$\begin{aligned} =2\times \frac{Precision\times Recall}{Precision+Recall} \end{aligned}$$

## Results and discussion

The Core i5 6th generation computer is used operating with Windows 10 64-bit, and 25 GB of RAM. Colab Pro GPU is used in this study to conduct the experiments. This section contains the complete experiments on the malaria cell-image dataset obtained from the Kaggle database. We used 11 deep-learning architectures with fine-tuned layers, one proposed model which consumes less energy and resources. The purpose of fine-tuning layers is to reduce the number of layers, choose dropout or dense layers according to the dataset, and make experiments more robust. We used a 32-bit batch size, 15 epochs, a 0.0001 learning rate, a categorical cross-entropy loss function, Adam, and the SGD optimizer. In addition, different train-test splits are used to analyze the performance of the models.

### Performance of fine-tuned deep learning models with 90:10 splitting

Table 4 shows the performance of fine-tuned deep learning models with 90:10 splitting for malarial cell data. The proposed approach achieved high performance in detecting malaria with 0.9750 accuracy, 0.9917 AUC, 0.9741 F1 score, and 0.9809 precision score. The achieved results by the proposed model are higher than those of other fine-tuned models. Also, the proposed model obtained a 0.1069 testing loss, which is the lowest when compared to others.

**Table 4:** Performance of fine-tuned deep learning models with 90:10 splitting.

Model	Accuracy	Precision	Recall	F1 score	AUC	Loss
DenseNet121	0.9470	0.9314	0.9652	0.9457	0.9849	0.1487
DenseNet201	0.9231	0.8881	0.9681	0.9245	0.9788	0.1977
DenseNet169	0.9481	0.9327	0.9659	0.9485	0.9827	0.1602
CNN	0.9586	0.9377	0.9826	0.9591	0.9786	0.2754
InceptionV3	0.8567	0.8206	0.9129	0.8592	0.9329	0.3737
VGG16	0.9507	0.9253	0.9804	0.9518	0.9706	0.4253
ResNet50	0.9184	0.8751	0.9761	0.9195	0.9738	0.2911
EfficientNet-B1	0.5522	0.5308	0.8991	0.6643	0.6124	0.6831
EfficientNet-B7	0.9630	0.9424	0.9862	0.9639	0.9903	0.1105
MobileNet	0.9521	0.9302	0.9775	0.9522	0.9789	0.2212
MobileNetV2	0.8817	0.8663	0.9028	0.8811	0.9533	0.2842
Proposed	0.9750	0.9809	0.9688	0.9741	0.9917	0.1069

The lowest accuracy is achieved by the EfficientNet-B1 model which is 0.5522, with the highest testing loss of 0.6831. The efficientNet-B7 achieved good accuracy and a 0.1105 testing loss. The second low-performing model is InceptionV3 with 0.8567 accuracy. VGG16 also achieved the highest testing loss, and its performance is not satisfactory.

### Performance of fine-tuned deep learning models with 80:20 splitting

The performance of fine-tuned deep learning models with 80:20 splitting for malarial cell data is shown in Table 5. When we used 80% of the data to train the model and 20% of the data to test it, the proposed model detected malaria with a 0.9757 accuracy score which is better than when a 90:10 split was used. The other metrics achieved are 0.9921 AUC, 0.9755 F1 score, and 0.9862 precision score. The achieved results by the proposed model are higher as compared to a 90:10 split. Also, the proposed model obtained a 0.0995 testing loss, which is very good. The EfficientNet-B1 model performed unsatisfactorily with this splitting and achieved a 0.6237 accuracy score. The highest testing loss is attained by the EfficientNet-B1 model, which is 0.6656. The second model that performs poorly is InceptionV3 with 0.8496 accuracy. Another VGG16 model achieved the highest testing loss.

**Table 5:** Performance of fine-tuned deep learning models with 80:20 splitting.

Model	Accuracy	Precision	Recall	F1 score	AUC	Loss
DenseNet121	0.9394	0.9285	0.9521	0.9389	0.9829	0.1618
DenseNet201	0.9115	0.8675	0.9713	0.9155	0.9754	0.2317
DenseNet169	0.9445	0.9259	0.9663	0.9442	0.9834	0.1712
CNN	0.9568	0.9393	0.9768	0.9564	0.9787	0.5021
InceptionV3	0.8496	0.8333	0.8741	0.8508	0.9114	0.4131
VGG16	0.9534	0.9334	0.9764	0.9540	0.9708	0.4507
ResNet50	0.9229	0.8820	0.9764	0.9262	0.9735	0.3079
EfficientNet-B1	0.6237	0.5922	0.7950	0.6734	0.6588	0.6656
EfficientNet-B7	0.9619	0.9517	0.9731	0.9620	0.9913	0.1108
MobileNet	0.9536	0.9417	0.9670	0.9530	0.9785	0.2442
MobileNetV2	0.8750	0.8666	0.8864	0.8740	0.9439	0.3172
Proposed	0.9757	0.9659	0.9862	0.9755	0.9921	0.0995

### K-fold cross validation results

K-fold cross-validation is the best method to evaluate the model’s robustness for classification, detection, and other problems. In this study, we divide the whole dataset into tenfolds, and this process is implemented using the K-fold class, TensorFlow, and Sklearn libraries. Table 6 shows the performance of various fine-tuned deep learning models with tenfold cross-validation. Experiments show that the proposed model is highly accurate in detecting malaria, with a 0.9724 accuracy score using a tenfold method. The proposed model has a recall score of 0.9847 and an AUC of 0.9872. The overall testing loss achieved by the proposed model is exceptionally low. Other deep learning models, EfficientNet-B1 and InceptionV3 fail to detect malaria with high accuracy. The EfficientNet-B7 also performed well under tenfold validation and achieved a 0.9539 accuracy score.

**Table 6:** Performance of fine-tuned deep learning models with tenfold cross-validation.

Model	Accuracy	Precision	Recall	F1 score	AUC	Loss
DenseNet121	0.9376	0.9319	0.9441	0.9377	0.9810	0.1686
DenseNet201	0.9238	0.9183	0.9303	0.9236	0.9735	0.2046
DenseNet169	0.9419	0.9319	0.9535	0.9400	0.9824	0.1854
CNN	0.9521	0.9469	0.9579	0.9512	0.9775	0.2152
InceptionV3	0.8443	0.8381	0.8533	0.8430	0.9296	0.3381
VGG16	0.9492	0.9322	0.9688	0.9500	0.9713	0.4528
ResNet50	0.9387	0.9501	0.9259	0.9375	0.9771	0.2585
EfficientNet-B1	0.5975	0.6143	0.5229	0.5591	0.6268	0.6772
EfficientNet-B7	0.9539	0.9516	0.9564	0.9534	0.9899	0.1247
MobileNet	0.9521	0.9456	0.9593	0.9517	0.9810	0.2239
MobileNetV2	0.8679	0.8700	0.8649	0.8647	0.9425	0.3221
Proposed	0.9724	0.9610	0.9847	0.9724	0.9872	0.1271

Table 7 shows the average performance of the proposed model. The proposed model achieved 0.9695 accuracy when we used twofold splitting; with threefold splitting, we achieved 0.9707 accuracy; as we increase the number of splits, accuracy increases. At ninefolds, we achieved 0.9758 accuracy, and at tenfolds, we achieved the highest accuracy of 0.9768. The proposed model has the lowest accuracy at fold 2 and the highest accuracy at fold 10. The precision, recall, F1 score, and AUC are 0.9663, 0.9802, 0.9724, and 0.9898, respectively.

**Table 7:** Average performance of the proposed model with K folds.

K-fold (n_splits)	Accuracy	Precision	Recall	F1 score	AUC
2	0.9695	0.9631	0.9765	0.9687	0.9906
3	0.9707	0.9668	0.9750	0.9705	0.9887
4	0.9727	0.9675	0.9782	0.9718	0.9911
5	0.9730	0.9670	0.9793	0.9722	0.9894
6	0.9723	0.9681	0.9769	0.9716	0.9917
7	0.9736	0.9707	0.9766	0.9731	0.9890
8	0.9724	0.9588	0.9872	0.9718	0.9903
9	0.9758	0.9637	0.9889	0.9751	0.9873
10	0.9768	0.9706	0.9833	0.9762	0.9903
Average	0.9730	0.9663	0.9802	0.9724	0.9898

### Testing accuracy and loss

**Figure 5:**
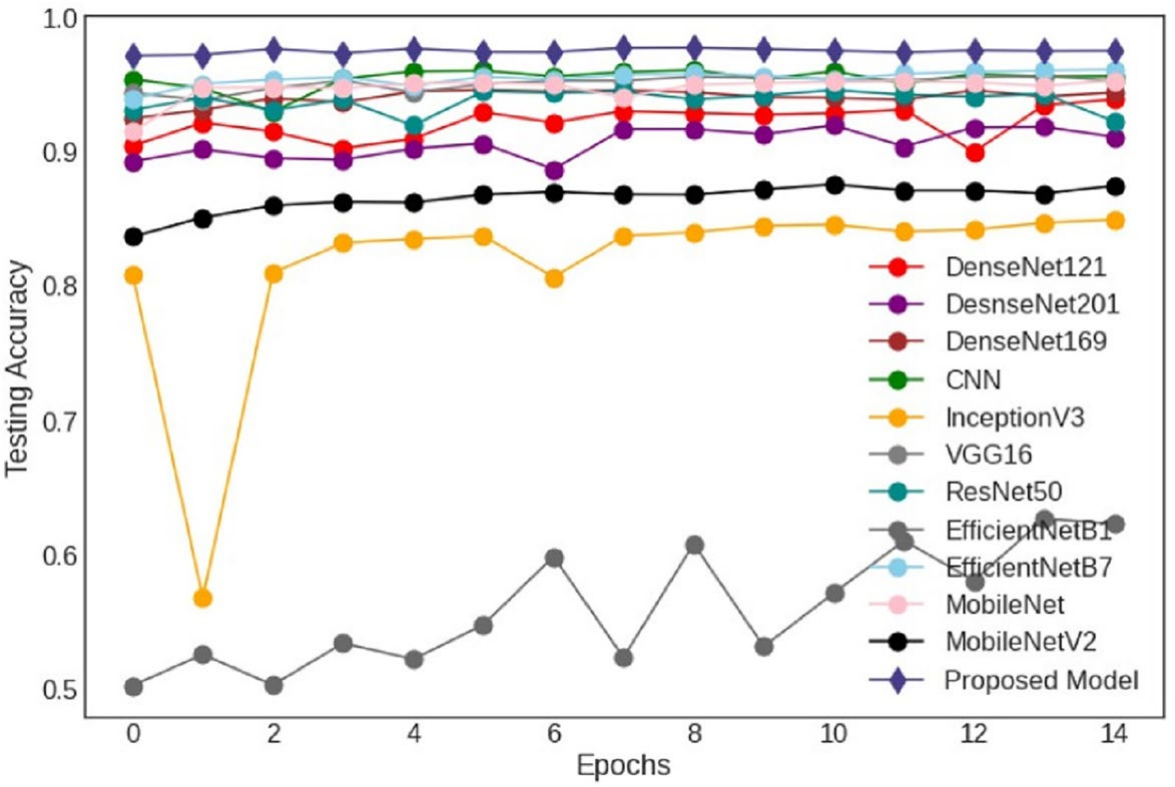
Testing accuracy of deep learning models.

The testing accuracy of proposed and various fine-tuned deep learning models is shown in Figure 5. It is observed that the EfficientNet-B1 curve is at the bottom, with the lowest accuracy at epoch 1, and after epoch 15, it reaches 62% testing accuracy. The other low-performing model is InceptionV3, where accuracy decreased abruptly at epoch 3 and then increased to exceed 84%. The proposed model curves rank first in terms of accuracy. There are little changes in the proposed model’s accuracy from epochs 1 to 15.

**Figure 6:**
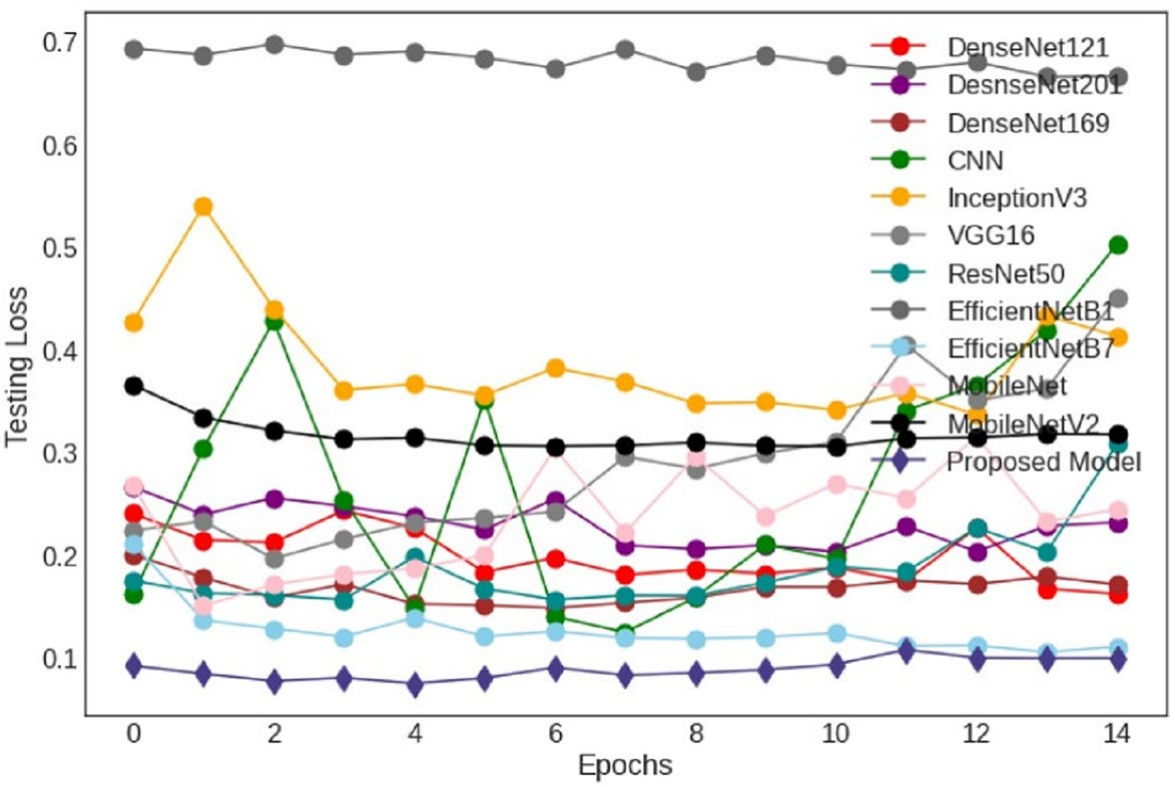
Testing loss of deep learning models.

Figure 6 shows the testing loss of various fine-tuned deep learning models. The experiment curves show that EfficientNet-B1 is at the top, with the highest testing loss. From epochs 1 to 15, the testing loss of EfficientNet-B1 has not decreased. The CNN model observed multiple variations at each epoch and loss at a higher stage at epoch 15. The CNN model’s testing loss is high, and this model cannot perform well. On the other -hand, the testing loss of our proposed model is excellent and very low. The proposed model testing loss is low as compared to other models.

### Confusion matrix results

We evaluated the model’s performance using an alternative validation dataset after training it. We used this set, distinguishable from the training data, to evaluate the model’s capacity to generalize to new samples. The evaluation involved the model making predictions on the validation set. We constructed the mathematical confusion matrix using the model’s predictions and the true labels from the validation dataset. We then applied a matrix arrangement to these counts.

**Figure 7:**
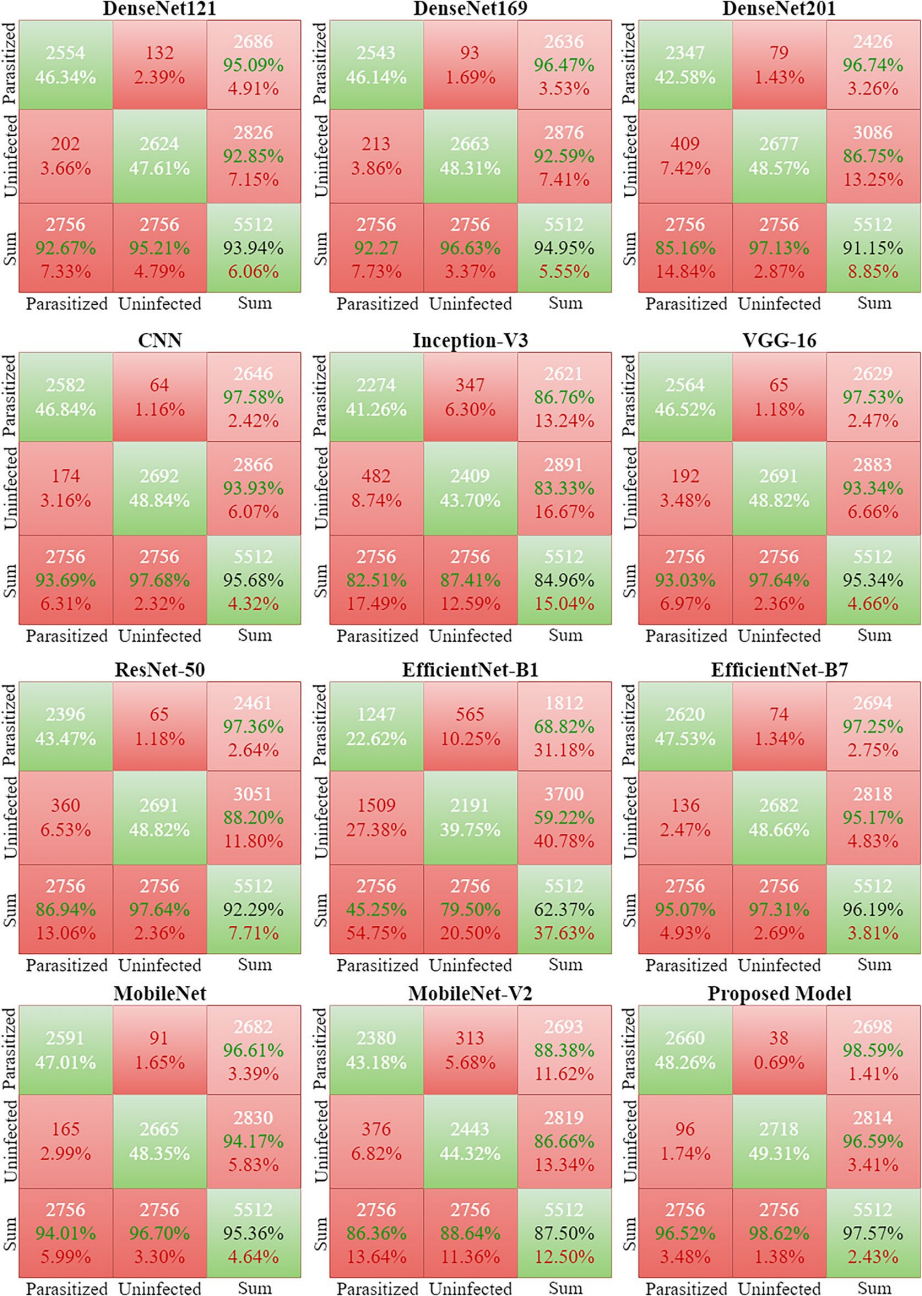
Results of deep learning and the proposed model regarding confusion matrix.

In Fig. 7, X-axis shows the predicted labels and Y-axis shows the true labels. We also calculated the sum for each class. For example, for the parasitized class, we calculated the sum on the X-axis and then the Y-axis. We also did the same for the uninfected class. In the confusion matrix, the white values or percentages indicate the true predictions, while the red values indicate the false predictions for both classes. Similarly, in the sum matrix, the white values indicate the total predictions, the green percentage indicates the true predictions, and the red percentage indicates the false predictions.

DenseNet121 has 5178 true predictions from a total of 5512 predictions. Results show that EfficientNetB1 performs badly, with 3438 true and 2074 false predictions. Another poor-performing deep model is InceptionV3, which achieved 4643 true predictions and 829 false predictions. All other deep fine-tuned models’ confusion matrix results prove that these models perform well with greater than 90% accuracy. The proposed approach achieved 5387 true and 134 false predictions.

**Table 8:** Proposed model comparison for statistical t-test operation scenario.

Comparison scenario	Statistical t test	P value	Hypothesis
Proposed vs DenseNet121	− 2.9361	0.0188	Reject null hypothesis
Proposed vs DenseNet201	− 2.4477	0.0400	Reject null hypothesis
Proposed vs DenseNet169	− 2.3919	0.0437	Reject null hypothesis
Proposed vs CNN	− 2.0258	0.0773	Reject null hypothesis
Proposed vs InceptionV3	− 8.0576	0.0000	Reject null hypothesis
Proposed vs VGG16	− 2.4305	0.0411	Reject null hypothesis
Proposed vs ResNet50	− 2.3533	0.0464	Reject null hypothesis
Proposed vs EfficientNet-B1	− 8.8944	0.0000	Failed to Reject null hypothesis
Proposed vs EfficientNet-B7	− 1.3607	0.2106	Reject null hypothesis
Proposed vs MobileNet	− 2.5947	0.0318	Reject null hypothesis
Proposed vs MobileNetV2	− 6.0877	0.0002	Reject null hypothesis

**Figure 8:**
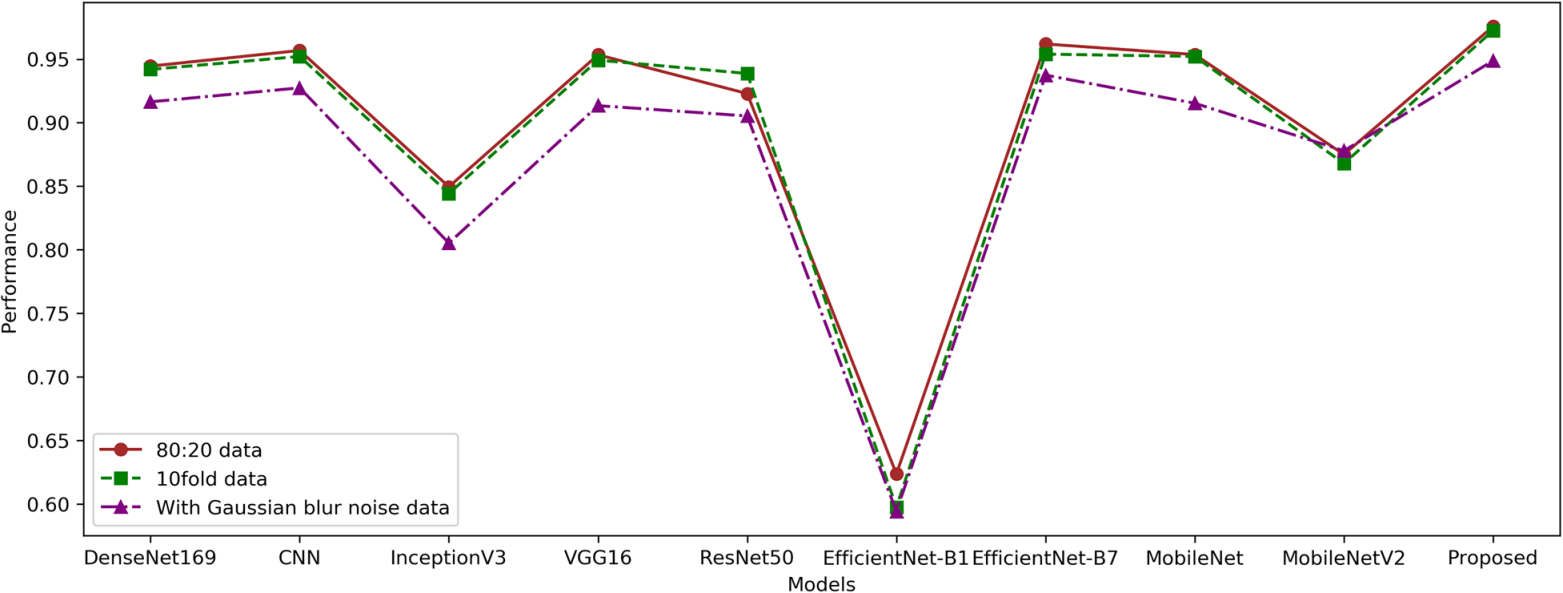
Comparison of the proposed model in various operation scenarios.

Proposed model comparison in statistical t-test operation scenario is presented in Table 8. For the statistical t-test, we employed a predetermined alpha, or significance level, when showing test results. The results demonstrate that the evidence is sufficient to reject the null hypothesis and establish a significant difference between the models when the p-value is less than the significance level (alpha = 0.05). However, if the p-value is greater than the significance level, it fails to reject the null hypothesis, which assumes that there is no significant difference among models. Figure 8 depicts the comparison results of the proposed model in various operation scenarios.

### Comparison with existing state-of-the-art models

We compare the results of the proposed approach to previously published work on malaria detection that used the same dataset. Accuracy, precision, recall, F1 score, and AUC are used to compare the effectiveness of the proposed approach. For example, Kalkan et al.^18^ used 27558 cell images for malaria detection through fine-tuned CNN and achieved a 95% accuracy score. Other performance metrics are not used in that study except accuracy. Similarly^20,21,24,26^, used CNN architecture with fine-tuned parameters on the same red blood-cell image dataset and achieved accuracy scores of 93%, 96%, 95.28%, and 95.70%, respectively. Furthermore, Vijayalakshmi and Rajesh Kanna^42^ employed a very small dataset of malaria cell images to detect the malaria using VGG-19+SVM model and results showed 93% accuracy and 91% F1 score. Hemachandran et al.^29^ identified malaria using CNN, MobileNetV2, and ResNet50. More accurately than others, MobileNetV2 performed better in the identification of malaria. With 27558 samples, they obtained an impressive accuracy of 97.06% and a superior AUC score of 96.77. Previous studies mostly utilized the CNN architecture to perform experiments on the same Malaria cell-image dataset. However, they detect malaria with low accuracy and high computing resources. Comparison results given in Table 9 show that the proposed approach achieved the highest accuracy and AUC in detecting malaria from red blood-cell images. The proposed approach achieved 99.21% AUC, 98.62% recall, and 97.57% accuracy.

**Table 9:** Comparison of the proposed model with existing state-of-the-art studies.

Reference	Method	No. of images	Accuracy	Precision	Recall	F1 score	AUC
^18^	Fine tuned CNN	27,558	95	–	–	–	–
^20^	CNN	27,558	93.72	–	–	–	–
^21^	Fast CNN	27,558	96	98	–	96	–
^23^	VGG16	27,558	96.15	94.82	97.54	96.16	–
^23^	2 layer-CNN	27,558	90.82	92.29	89.08	90.66	–
^43^	DCNN	27,560	95.23	95	95	95	–
^26^	CNN	27,558	95.70	96	96	96	–
^24^	CNN	27,558	95.28	95.1	95.5	–	–
^28^	DACNN	27,558	94.79	–	–	–	–
^42^	VGG19+SVM	2550	93.1	89.95	93.44	91.66	–
^29^	MobileNetV2	27,558	97.06	97	97	98	96.73
Proposed		27,558	97.57	96.59	98.62	97.55	99.21

## Conclusion

This study proposes an automated, Efficient model for malaria parasite detection from red blood cell images. The traditional methods to detect malaria are not efficient, provide low accuracy, and require higher computational time. The proposed model achieved the highest accuracy score of 97.57% and the highest AUC score of 99.21%. The other pre-trained and fine-tuned deep learning models achieved poor classification accuracy and recall scores. The confusion matrix resulting from the proposed model predicts 2660 correct predictions and only 38 wrong predictions from a total of 2698 predictions for the parasitized class and 2718 correct predictions for the uninfected class. The proposed model has a 98.59% accuracy for the parasitized class. K-fold cross-validation and performance comparison with existing state-of-the-models show the superiority and robustness of the proposed approach. In the future, we intend to enlarge the red blood smear dataset from different repositories and develop a comprehensive system for malaria detection with parallel computing devices to minimize the training time.

## Data Availability

The datasets used and/or analysed during the current study available at https://ceb.nlm.nih.gov/repositories/malaria-datasets/.
